# Assessment of age-at-onset criterion for adult attention-deficit hyperactivity disorder

**DOI:** 10.1192/bjp.2021.122

**Published:** 2021-09-21

**Authors:** Lucy Riglin, Rachel Blakey, Kate Langley, Ajay K Thapar, Sharifah Shameem Agha, George Davey Smith, Evie Stergiakouli, Anita Thapar

**Affiliations:** 1 Division of Psychological Medicine and Clinical Neurosciences, MRC Centre for Neuropsychiatric Genetics and Genomics, Cardiff University, UK; 2 Population Health Sciences and MRC Integrative Epidemiology Unit, University of Bristol, Bristol, UK; 3 School of Psychology, Cardiff University, Wales, UK

**Keywords:** ADHD, adult, age-at-onset, retrospective, ALSPAC

## Abstract

To investigate the accuracy of the age-at-onset criterion in those who meet other DSM-5 ADHD criteria (N=138), using a prospective population cohort, we compared four different approaches to asking those at age 25 years when their symptoms started. Receiver Operating Characteristic curves showed variation between the approaches (χ_(3)_)=8.99, p=0.03); all four showed low discrimination against symptoms that had been assessed when they were children (area under the curve 0.57-0.68). Asking adults to recall specific symptoms may be preferable to recalling at what age symptoms started. However limitations to retrospective recall add to debate on the validity of ADHD age-at-onset assessment.

## Assessment of age-at-onset criterion for adult attention-deficit hyperactivity disorder

One criterion required for a diagnosis of ADHD is symptom onset before age 12 years (1). When individuals first present to clinicians as adults, this requires retrospective recall of symptoms and likely limits accuracy (1, 2) due to both false-positives and false-negatives (3). Identifying the optimal method to assess ADHD age-at-onset is an important question for adult psychiatrists. We compared the accuracy of four different ways to assess ADHD age-at-onset in a prospective population cohort. We focus on those who met the other DSM-5 criteria for ADHD at age 25 years: at-least five inattentive or five hyperactive/impulsive symptoms plus impairment.

## Method

We analysed data from the Avon Longitudinal Study of Parents and Children (ALSPAC) (4) which includes repeated assessments since pregnancy (see Supplementary Material). Ethical approval was obtained from the ALSPAC Law and Ethics Committee and Local Research Ethics Committees. Informed consent was obtained from participants following the recommendations of the ALSPAC Ethics and Law Committee at the time.

138 (42% male) individuals met DSM-5 symptom and impairment criteria at age 25 with complete data on age-at-onset and ADHD symptoms assessed in childhood (see below).

### Age 25 assessment

DSM-5 symptom and impairment criteria were assessed using self-reports of the Barkley Adult ADHD Rating Scale (BAARS-IV)(5, 6). Parents also completed the BAARS-IV: these data were used for sensitivity analyses (see below).

The BAARS-IV uses two sets of questions for age-at-onset – (a) *specify age:* individuals were asked to recall as precisely as possible at what age these problems (ADHD symptoms) began to occur (in years) and (b) *rate behaviour between 7 and 12 years:* individuals were asked to rate the frequency of 18 DSM-5 ADHD symptoms on a 4-point scale.

We generated four retrospective definitions of ADHD age-at-onset before age 12 years:

- *Specified age* that ADHD symptoms began to occur was before age 12 years.
- *At-least one ADHD symptom* was endorsed as having been clinically significant (occurring ‘often’ or ‘very often’ (6)) between 7 and 12 years.
- *Several symptoms* (at-least three) were endorsed as having been clinically significant between 7 and 12 years (DSM-5 requires ‘several’ inattentive or hyperactive/impulsive symptoms present prior to age 12 years (7)).
- *At least six inattentive or six hyperactive/impulsive symptoms* were endorsed as having been clinically significant between 7 and 12 years (DSM-5 symptom requirement for childhood ADHD (7)).

### ADHD symptoms assessed during childhood

These had been assessed when these adults were aged 7, 8, 9 and 12 years using the 5-item ADHD subscale of the Strengths and Difficulties Questionnaire (SDQ)(8) rated by parents, as children’s self-reports are unreliable (9). The SDQ is a screening questionnaire with symptoms in the past 6 months categorised as low (0-5), slightly raised (6-7) or high (8-10)(8). Individuals with slightly raised or high symptoms (≥6) at any of these ages were defined as having ADHD symptoms when assessed in childhood: this was used to test the accuracy of adult retrospective reports of age-at-onset. This broad definition was used given the DSM-5 requirement that ‘several’ symptoms present prior to age 12 years (7).

### Measures for sensitivity analyses

(a) ADHD assessed during childhood defined based on full ADHD diagnosis at age 7/10 years, measured using the parent-rated Development and Well-Being Assessment (9) (described in the Supplementary Materials), (b) age 25 assessments of age-at-onset using the parent-rated BAARS-IV.

### Analyses

Receiver Operating Characteristic (ROC) curve analyses using Stata’s *roccomp* function were used to examine the validity of the four retrospective assessments of ADHD age-at-onset in distinguishing those with versus those without ADHD symptoms when assessed in childhood.

## Results

Of those who met DSM-5 criteria for adult ADHD symptoms and impairment (N=138) at age 25, when asked to specify the age at which symptoms onset 51% (N=71) reported onset before age 12 years. When asked to rate behaviour between 7 and 12 years, 86% (N=119) retrospectively reported at-least one ADHD symptom, 72% (N=100) reported at-least three symptoms and 44% (N=61) retrospectively reported six inattentive and/or six hyperactive/impulsive symptoms.

Results for the four ADHD age-at-onset assessments are shown in Table 1. All approaches showed low discrimination in identifying ADHD symptoms assessed in childhood (AUC=0.57-0.68), although there was evidence that this varied across the four approaches (χ_(3)_=8.99, p=0.03).

Reporting at-least one symptom showed the highest sensitivity (the proportion of those with symptoms when assessed in childhood correctly identified by retrospective reports) and negative predictive validity (NPV: the proportion of those retrospectively reported not to have childhood-onset correctly identified) and the lowest specificity (the proportion of those without symptoms when assessed in childhood correctly identified by retrospective reports) and positive predictive validity (PPV: the proportion of those retrospectively identified who did have symptoms when assessed in childhood). Conversely retrospectively endorsing at-least six inattentive or six hyperactive/impulsive childhood symptoms showed the highest specificity and PPV whereas specifying age showed the lowest sensitivity and NPV.

### Sensitivity analyses

Sensitivity analyses where ADHD assessed in childhood was defined based on full diagnostic criteria are shown in Supplementary Table 1 (N=122): this showed a similar pattern of results although with somewhat higher discrimination (AUC=0.60-0.81: χ_(3)_=96.00, p=1x10^-20^). Parent retrospective reports of age-at-onset at age 25, shown in Supplementary Table 2 (N=47): this showed fairly low discrimination (AUC=0.63-0.70) with little evidence of variation across the four approaches (χ_(3)_=1.19, p=0.76).

## Discussion

We found variation in the discrimination of four approaches to retrospectively assess ADHD age-at-onset at age 25 years; although all showed limited validity. This is consistent with a Brazilian birth-cohort findings (10). Of the four approaches, the highest proportion of participants met age-at-onset criteria when this was defined based on asking participants to retrospectively rate their behaviour between ages 7 and 12 years, and requiring the endorsement of at-least one of the 18 DSM ADHD symptoms: this definition (which does not fit with the DSM-5 requirement that ‘several’ symptoms present prior to age 12 years) resulted in the highest proportion of true positives (highest sensitivity) but also the fewest true negatives. Conversely the highest specificity (and lowest proportion of people identified) was found using the most stringent definition: the retrospective endorsement of at-least six inattentive and/or six hyperactive/impulsive childhood symptoms.

The alternative approach of asking participants to specify the age at which endorsed symptoms started resulted in the fewest true positives, i.e. this missed the most people who had ADHD symptoms when assessed in childhood. This provides tentative evidence that asking people to recall specific symptoms during a specific age period is preferable to recalling the age at which symptoms started. However none of the four approaches showed high accuracy, which is consistent with previous work highlighting the limitations of retrospective recall (3). Sensitivity analyses defining ADHD assessed in childhood based on full DSM-5 diagnostic criteria (and requiring the retrospective endorsement of six inattentive and/or six hyperactive/impulsive childhood symptoms) showed moderate discrimination. This suggests that recall of more severe and impairing symptoms may be better than for just a few symptoms. In practice there is likely benefit in asking about specific ADHD symptoms in childhood and acquiring additional information from other sources, e.g. school reports.

While the age-at-onset criterion for ADHD is important from a developmental perspective (1), our results, alongside increasing evidence of “late-onset” ADHD (2), raise queries about its validity. Further research is needed to address the limitations of the current work, including limited sample size and non-random attrition. Defining age-at-onset is important for informing adult psychiatrists and diagnostic criteria.

## Supplementary Material

Supplementary Material

## Figures and Tables

**Table 1:** Discrimination of retrospective assessments of ADHD age-at-onset criterion in distinguishing those with and without ADHD symptoms when assessed in childhood, in youngadults with ADHD symptoms and impairment at age 25 years

	ROC AUC (95% CI)	Accuracy	Sensitivity	Specificity	PPV	NPV
Specified age	0.60 (0.52-0.69)	60%	63%	58%	55%	66%
At-least one symptom	0.57 (0.51-0.62)	53%	94%	20%	49%	79%
At least three (several) symptoms	0.62 (0.55-0.69)	59%	76%	53%	53%	76%
Six inattentive and/or six hyperactive/impulsive symptoms	0.68 (0.61-0.76)	69%	65%	72%	66%	71%

ROC = Receiver Operating Characteristic, AUC = area under the curve, PPV = positive predictive values, NPV = negative predictive values.

## Data Availability

The ALSPAC data management plan (http://www.bristol.ac.uk/alspac/researchers/data-access/documents/alspac-data-management-plan.pdf) describes in detail the policy regarding data sharing, which is through a system of managed open access.
