# Supplementary material for: Assessment of age-at-onset criterion for adult attention-deficit hyperactivity disorder
Source: Br J Psychiatry. Author manuscript; Available in PMC 2022 Feb 1. (PMC7612274; doi:10.1192/bjp.2021.122)
Supplement: Supplementary Material [file EMS132495-supplement-Supplementary_Material.pdf]

# Supplementary Material

## The Avon Longitudinal Study of Parents and Children (ALSPAC)

Pregnant women resident in Avon, UK with expected dates of delivery 1st April 1991 to 31st December 1992 were invited to take part in the study. The initial number of pregnancies enrolled is 14,541 (for these at least one questionnaire has been returned or a "Children in Focus" clinic had been attended by 19/07/99). Of these initial pregnancies, there was a total of 14,676 foetuses, resulting in 14,062 live births and 13,988 children who were alive at 1 year of age. When the oldest children were approximately 7 years of age, an attempt was made to bolster the initial sample with eligible cases who had failed to join the study originally. As a result, the total sample size for data collected after the age of seven is therefore 15,454 pregnancies, resulting in 15,589 foetuses. Of these 14,901 were alive at 1 year of age. Where families included multiple births, we included the oldest sibling. Further details of the study, measures and sample can be found elsewhere (1-3). The number of individuals with sufficient data for inclusion in our analyses are shown in Supplementary Figure 1.

Part of these study data were collected and managed using REDCap electronic data capture tools hosted at the University of Bristol(4). REDCap (Research Electronic Data Capture) is a secure, web-based software platform designed to support data capture for research studies. Please note that the study website contains details of all the data that is available through a fully searchable data dictionary and variable search tool: http://www.bristol.ac.uk/alspac/researchers/our-data/. Ethical approval for the study was obtained from the ALSPAC Law and Ethics Committee and Local Research Ethics Committees. Informed consent for the use of data collected via questionnaires and clinics was obtained from participants following the recommendations of the ALSPAC Ethics and Law Committee at the time.

## Childhood assessments of ADHD diagnosis

ADHD was assessed at ages 7- and 10-years using parent reports of the Development and Well-Being Assessment (5), a well-established research diagnostic assessment. DSM-IV ADHD diagnosis were generated through computer algorithms (6), whereby children were defined as having a diagnosis if they were in the highest two computer predicted band (50% of children in this band predicted to have disorder). Individuals with a diagnosis at either age were coded as meeting full diagnostic criteria for ADHD in childhood (9/122: 7%, all of whom had ADHD symptoms when assessed in childhood as defined for our primary analyses). DAWBA assessments were included as a sensitivity, rather than primary measure of ADHD symptoms

during childhood because data were collected at fewer time-points (two time-points) compared to the Strengths and Difficulties Questionnaire (four time-points), thus giving a less complete assessment of the presence of childhood symptoms.

**Supplementary Table 1**. Discrimination of retrospective assessments of ADHD age-at-onset criterion in distinguishing those with and without ADHD diagnosis when assessed in childhood, in young-adults with ADHD symptoms and impairment at age 25 years

| | ROC AUC (95% CI) | Accuracy | Sensitivity | Specificity | PPV | NPV |
|---|---|---|---|---|---|---|
| Specified age | 0.71 (0.59-0.83) | 56% | 89% | 53% | 13% | 98% |
| At-least one symptom | 0.60 (0.55-0.61) | 22% | 100% | 16% | 9% | 100% |
| At least three (several) symptoms | 0.65 (0.61-0.70) | 36% | 100% | 31% | 10% | 100% |
| Six inattentive and/or six hyperactive/impulsive symptoms | 0.81 (0.76-0.85) | 65% | 100% | 62% | 17% | 100% |

ROC = Receiver Operating Characteristic, AUC = area under the curve, PPV = positive predictive values, NPV = negative predictive values.

**Supplementary Table 2**. Discrimination of retrospective assessments of parent-rated ADHD age-at-onset criterion in distinguishing those with and without ADHD symptoms when assessed in childhood, in young-adults with ADHD symptoms and impairment at age 25 years

| | ROC AUC (95% CI) | Accuracy | Sensitivity | Specificity | PPV | NPV |
|---|---|---|---|---|---|---|
| Specified age | 0.63 (0.51-0.76) | 64% | 83% | 43% | 61% | 71% |
| At-least one symptom | 0.70 (0.57-0.84) | 70% | 71% | 70% | 71% | 70% |
| At least three (several) symptoms | 0.68 (0.55-0.82) | 68% | 63% | 74% | 71% | 65% |
| Six inattentive and/or six hyperactive/impulsive symptoms | 0.69 (0.58-0.80) | 68% | 42% | 96% | 91% | 61% |

ROC = Receiver Operating Characteristic, AUC = area under the curve, PPV = positive predictive values, NPV = negative predictive values.

**Supplementary Figure 1**. Individuals with sufficient data for inclusion in the analyses

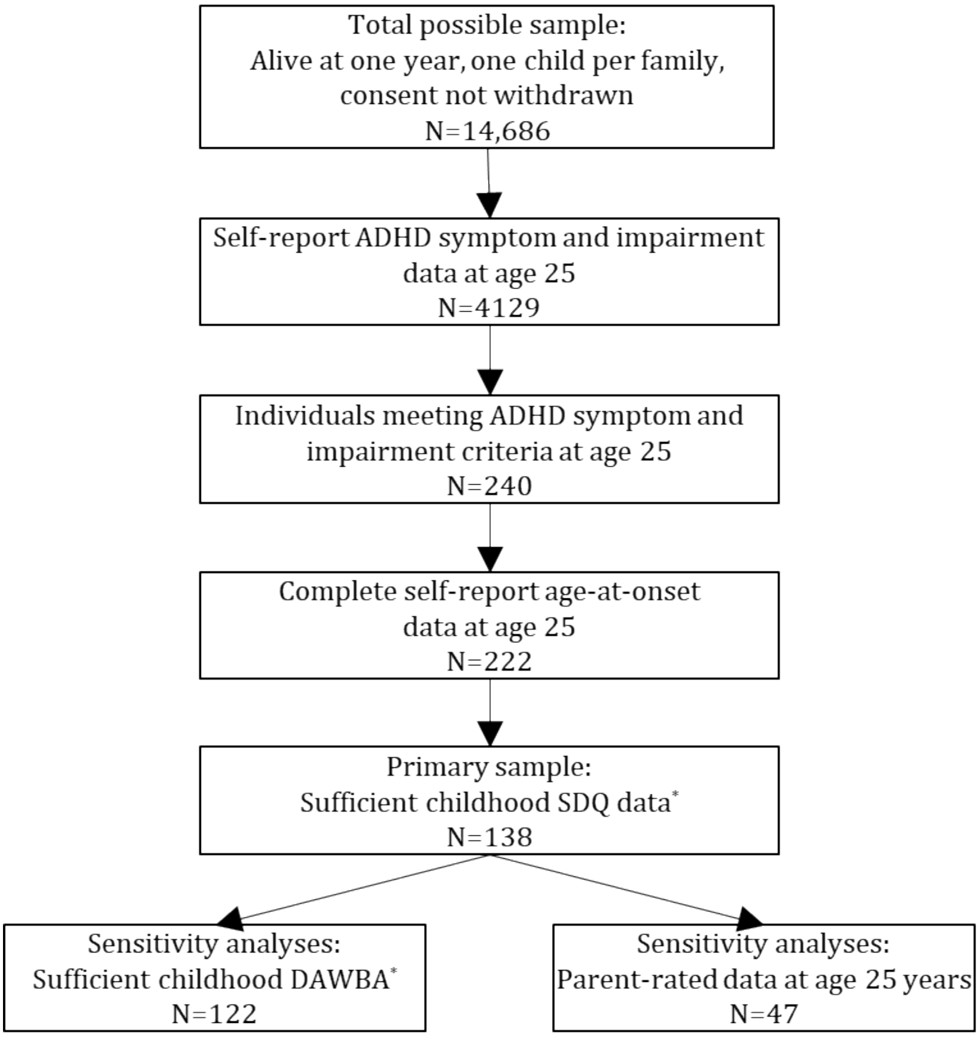


SDQ = ADHD sub-scale of the Strengths and Difficulties Questionnaire, DAWBA = ADHD section of the Development and Well-Being Assessment. *Symptoms present at any assessment or absent at every assessment.

**References**

1. Boyd A, Golding J, Macleod J, Lawlor DA, Fraser A, Henderson J, et al. Cohort Profile: the 'children of the 90s'--the index offspring of the Avon Longitudinal Study of Parents and Children. International journal of epidemiology. 2013; 42(1): 111-27.
2. Fraser A, Macdonald-Wallis C, Tilling K, Boyd A, Golding J, Davey Smith G, et al. Cohort Profile: the Avon Longitudinal Study of Parents and Children: ALSPAC mothers cohort. International journal of epidemiology. 2013; 42(1): 97-110.
3. Northstone K, Lewcock M, Groom A, Boyd A, Macleod J, Timpson N, et al. The Avon Longitudinal Study of Parents and Children (ALSPAC): an update on the enrolled sample of index children in 2019. Wellcome Open Res. 2019; 4: 51-.
4. Harris PA, Taylor R, Thielke R, Payne J, Gonzalez N, Conde JG. Research electronic data capture (REDCap)--a metadata-driven methodology and workflow process for providing translational research informatics support. J Biomed Inform. 2009; 42(2): 377-81.
5. Goodman R, Ford T, Richards H, Gatward R, Meltzer H. The Development and Well-Being Assessment: description and initial validation of an integrated assessment of child and adolescent psychopathology. J Child Psychol Psychiatry. 2000; 41(5): 645-55.
6. Goodman A, Heiervang E, Collishaw S, Goodman R. The 'DAWBA bands' as an ordered-categorical measure of child mental health: description and validation in British and Norwegian samples. Soc Psychiatry Psychiatr Epidemiol. 2011; 46(6): 521-32.
